# Molecular Genetic Analysis of Bone Marrow Core Biopsy as an Alternative or Adjunct to Bone Marrow Aspirate and/or Peripheral Blood in Hematologic Myeloid Neoplasms

**DOI:** 10.3390/diagnostics15080991

**Published:** 2025-04-14

**Authors:** Klaus Hirschbühl, Bruno Märkl, Gernot Müller, Tina Schaller, Rainer Claus, Sebastian Sommer, Maximilian Schmutz, Martin Trepel, Christoph Schmid, Sebastian Dintner

**Affiliations:** 1 Hematology and Oncology, Medical Faculty, University of Augsburg, 86156 Augsburg, Germany; rainer.claus@uk-augsburg.de (R.C.); s.sommer@onkologie-elisenhof.de (S.S.); maximilian.schmutz@uk-augsburg.de (M.S.); martin.trepel@uk-augsburg.de (M.T.); christoph.schmid@uk-augsburg.de (C.S.); 2 Bavarian Center for Cancer Research (BZKF), 86156 Augsburg, Germany; bruno.maerkl@uk-augsburg.de (B.M.); tina.schaller@uk-augsburg.de (T.S.); sebastian.dintner@uk-augsburg.de (S.D.); 3 Pathology, Medical Faculty, University of Augsburg, 86156 Augsburg, Germany; 4 Department of Computational Statistics and Data Analysis, University of Augsburg, 86159 Augsburg, Germany; gernot.mueller@uni-a.de; 5 Onkologie/Hämatologie im Elisenhof, 80335 München, Germany

**Keywords:** bone marrow core biopsies, formalin-fixed paraffin-embedded, myeloid neoplasm, next generation sequencing

## Abstract

**Background**: The diagnosis of hematologic neoplasms is usually based on a synopsis of the peripheral blood (PB) and bone marrow findings. Morphology continues to be the cornerstone, but genetic analysis plays an increasingly important role. In routine workup, molecular genetic analysis is performed from a bone marrow aspirate (BMA). In the event of inadequate aspiration, PB is used. Not infrequently, however, PB only partially represents the disease. In this situation, molecular genetic analysis of formalin-fixed and paraffin-embedded (FFPE) bone marrow core biopsy (BMCB) could be a better alternative than PB. However, no systematic correlation of genetic findings from BMCB with results from BMA and PB has been reported. **Methods**: Therefore, BMCB obtained during routine diagnostics were subjected to post hoc molecular genetic analysis using next generation sequencing (NGS). The identified molecular genetic alterations were then compared with data within routine diagnostics of the corresponding BMA and/or PB. **Results**: In total, 29 BMCB and corresponding BMA samples were analyzed, and in 12/29 cases PB was additionally available. The analysis of BMCB and BMA showed identical results in 17 cases, but BMCB revealed a gain of information in 11, while in only 1 case, BMCB failed to identify the mutations in comparison to BMA. **Conclusions**: Despite the small numbers, molecular genetic analysis of bone marrow core biopsy using next generation sequencing could detect relevant additional gene mutations compared to bone marrow aspirate and/or peripheral blood.

## 1. Introduction

Hematologic, and especially myeloid, neoplasms are usually diagnosed from a synopsis of peripheral blood and bone marrow findings. Morphology continues to be the basis of this, but genetics and especially molecular genetic analysis, are now becoming more important. Thus, some (sub)entities are nowadays mainly defined genetically, or the genetic markers play an essential role in risk classification and/or even have relevant therapeutic implications, which is reflected in the current classifications of both the World Health Organization and the International Consensus Classification published in 2022 [1,2,3]. In the diagnosis of hematologic myeloid neoplasms, the examination of the bone marrow, both morphologically and genetically, plays a central role [4,5]. In addition to classical cytogenetics, molecular genetics has become an established component of this genetic diagnostics. For technical reasons alone, molecular genetic testing has so far been performed exclusively on bone marrow aspiration (BMA), but not on bone marrow core biopsies (BMCB). Peripheral blood (PB) next-generation sequencing (NGS) testing recently showed a high concordance to BMA, but is only considered as an alternative if the disease is represented in the peripheral blood to a relevant extent [6,7,8]. However, this is not given in every case. In the situation of an insufficient bone marrow aspiration (no bone marrow fragments in the aspirate, only blood = bloody tap, included in this study; no aspiration possible = dry tap, not included in this study), genetic analysis is often not possible at all in case of a dry tap or could be inadequate in case of a bloody tap. Therefore, molecular genetic analysis of BMCB on formalin-fixed and paraffin-embedded (FFPE) material would be a possibly better alternative compared to PB in the case of non-representative BMA. This is already performed in a standardized manner on FFPE tissues from solid tumors and no longer presents technical challenges, as the panel designs have been adapted according to the requirements of the FFPE material. Only a few reports described NGS on FFPE BMCB [9], but a comparison of BMA, BMCB and PB has not been described so far.

The aim of this pilot study is to technically establish the molecular genetic analysis of FFPE BMCB using NGS with a myeloid gene panel and to evaluate and validate it by comparison to BMA and PB.

## 2. Materials and Methods

### 2.1. Study Collection and Specimens

This study collection is retrospective and assembled on the basis of the following inclusion criteria: (1) myeloid neoplasm, (2) present molecular genetic analysis from BMA (including bloody tap = no bone marrow fragments in the aspirate, only blood) and/or PB already performed for routine diagnostics (last inclusion by 11/2022 at the latest), and (3) the availability of BMCB stored as FFPE taken for routine diagnostic. Then, BMCB was analyzed for molecular genetic alterations on the same platform on which it had been performed for BMA and/or PB for the routine diagnostics before. Specimens could be taken at first diagnosis of the disease or in follow-up examinations.

BMCB are archived as FFPE material (12 h fixation in formaldehyde solution, dehydration and decalcification via Logos J^®^ from Milestone (Valbrembo, Italy), embedding the material into paraffin) at the Institute of Pathology and Molecular Diagnostics at Augsburg University Hospital for assessment of routine morphological and immunohistological diagnostics. Molecular genetic analysis of BMA and PB samples was performed by molecular pathology in collaboration with hematology at the Augsburg University Hospital, and also as part of routine diagnostics. Molecular genetic analysis for PB and BMA was performed routinely within a few days after the collection of the sample. The analysis of BMCB was performed after including the case in the study collection.

The study was approved by the ethics committee of the Ludwig-Maximilians-University of Munich (reference number: 22-1043; approval date: 10 January 2023).

### 2.2. DNA and RNA Isolation, Next-Generation Sequencing (NGS)

The Maxwell^®^ RSC Whole Blood DNA Kit and the Maxwell^®^ RSC simplyRNA Blood Kit (Promega, Madison, WI, USA) were used to isolate DNA and RNA from peripheral blood samples and BMA. For isolation from BMCB, the Maxwell^®^ RSC FFPE Plus DNA Kit and the Maxwell^®^ RSC RNA FFPE Kit was utilized. Relevant regions of the genome were enriched by targeted amplicon-based enrichment (NGS) using the AmpliSeq for Illumina Myeloid Panel (Illumina, San Diego, CA, USA) and sequenced with the MiSeq System (Illumina) using the V2 (500) chemistry (Illumina) for 8 samples per run. This involves the analysis of the coding exons of 70 tumor-related genes for point mutations, deletions, and insertions, and also gene fusions at the RNA level. This has already been performed routinely on conventional BMA and PB under accredited conditions since 2019. For the study, the Institute of Pathology and Molecular Diagnostics archived the associated BMCB. In these conditions, the identical panel was used for molecular alignment.

Sequencing was performed with an average coverage of ≥1000 using Illumina MiSeq technology. Data analysis was performed using the Illumina LRM application DNA Amplicon and subsequent interpretation using a Variant Interpreter (Illumina), in which filtering was performed for non-synonymous and non-polymorphic alterations. The limit of detection (LOD) of variant allele frequency was 1%. In addition, the data were analyzed using the BaseSpace Knowledge Network and the variants were documented accordingly. Interpretation of the changes was performed using different databases, such as cbioportal.org, ClinVar, Varsome, and CiVIC.

### 2.3. Statistical Analysis

Descriptive statistics were performed to demonstrate the detected mutated genes and their variant allele frequency (VAF) and to compare the absolute number of mutations detected within the three different specimens (PB, BMA, and BMCB). Due to the fact that it is possible to harbor more than one mutation within one gene, it could be that in BMA, BMCB, and PB, in one case the number of detected mutations is the same, but different mutated genes or different mutations within one gene. Given this fact, comparing only the number of mutations might not be sufficiently accurate. Therefore, an additional categorization with concordance, gain, or loss of information was performed to compare BMA, BMCB, and PB within one case.

Statistical testing for detecting differences between BMA and BMCB was performed. McNemar’s chi-squared test was used to test for the difference with regard to concordance, gain or loss of information. Due to the small numbers of PB, testing for statistical significance was not intended. All statistical analyses were performed using SPSS 29.02.0 (IBM Corp., Armonk, NY, USA).

## 3. Results

### 3.1. Description of the Collection and Specimens

**Figure 1:**
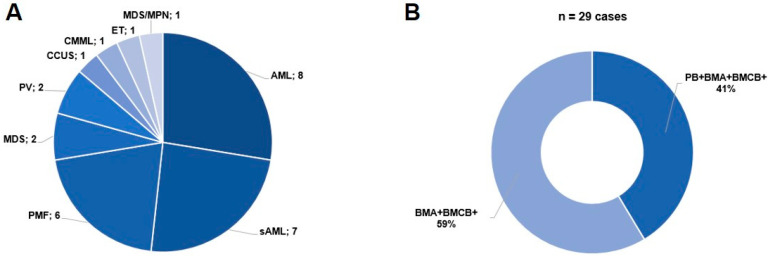
Summary of collection. (**A**): Distribution of entities among *n* = 29 cases. (**B**): Available specimens. Abbreviations: (s)AML: (secondary) acute myeloid leukemia, PMF: primary myelofibrosis, MDS: myelodysplastic syndrome, PV: polycythemia vera, CCUS: clonal cytopenia of undetermined significance, CMML: chronic myelomonocytic leukemia, ET: essential thrombocytosis, MDS/MPN: myelodysplastic syndrome/myeloproliferative neoplasia, PB: peripheral blood, BMA: bone marrow aspiration, BMCB: bone marrow core biopsy.

A total of 29 cases were identified that met the inclusion criteria. In 17 of the 29 cases, only BMA and BMCB, and in 12 of the 29 cases, molecular genetic analysis of all three specimens, BMA, BMCB, and PB, were available. The entities were acute myeloid leukemia (AML), secondary acute myeloid leukemia (sAML), primary myelofibrosis (PMF), myelodysplastic syndrome (MDS), polycythemia vera (PV), clonal cytopenia of undetermined significance (CCUS), chronic myelomonocytic leukemia (CMML), essential thrombocytopenia (ET), and myelodysplastic/myeloproliferative neoplasm, not otherwise specified (MDS/MPN-NOS). For the distribution of the entities and available specimens, see Figure 1. Bloody tap was evident in 16 out of the 29 cases. BMA and BMCB samples were taken on the same day in all but one case. In this case, there was a delay of 39 days, but no specific therapy had been applied in between. Due to the retrospective nature of the study, the median time between BMA/BMCB and PB sampling was 0 days (range 0–39). In some cases, PB was only taken and analyzed when this was considered clinically necessary (e.g., bloody tap and/or diagnosis of myeloproliferative neoplasms (MPN), especially PMF).

### 3.2. DNA and RNA Quality Assessment and AmpliSeq Performance

**Figure 2:**
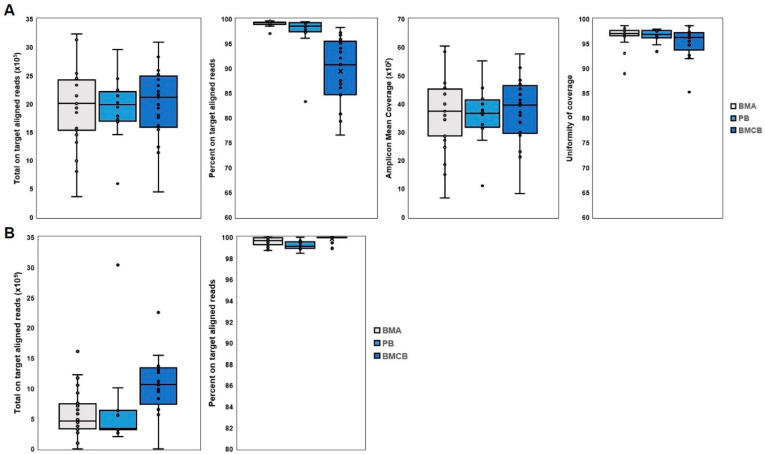
Quality control parameters for DNA and RNA sequencing analysis. (**A**): DNA sequencing analysis: box plots displaying the QC parameters for DNA sequencing across three sample types. (**B**): RNA sequencing analysis: box plots showing the QC parameters for RNA sequencing across the same three sample types: BMA (gray), PB (light blue), and BMCB (dark blue). For both DNA and RNA sequencing, PB and BMCB samples generally demonstrate higher consistency and less variability compared to BMA samples. The central lines in the boxes represent the medians, the edges of the boxes denote the IQRs, and the whiskers extend to 1.5 times the IQR. Each sample is represented by individual points. Abbreviations: QC: quality control, BMA: bone marrow aspiration, PB: peripheral blood, BMCB: bone marrow core biopsy, IQR: interquartile range.

In our quality control analysis of DNA and RNA sequencing, we observed high overall performance across all sample types. For DNA sequencing (Figure 2A), BMA, PB, and BMCB samples exhibited high median values for total on-target aligned reads, percent on-target aligned reads, amplicon mean coverage, and uniformity of coverage. However, BMA and PB samples demonstrated more consistent results with tighter distributions and less variability compared to BMCB samples. In RNA sequencing (Figure 2B), we found similarly high median percentages of on-target aligned reads across all sample types, with PB and BMCB showing higher and more consistent total on-target aligned reads than BMA. These findings indicate that while all sample types generally performed well, PB and BMA samples provided more reliable and uniform sequencing metrics compared to BMCB samples.

### 3.3. Molecular Genetic Analysis and Detection of Mutated Genes

**Figure 3:**
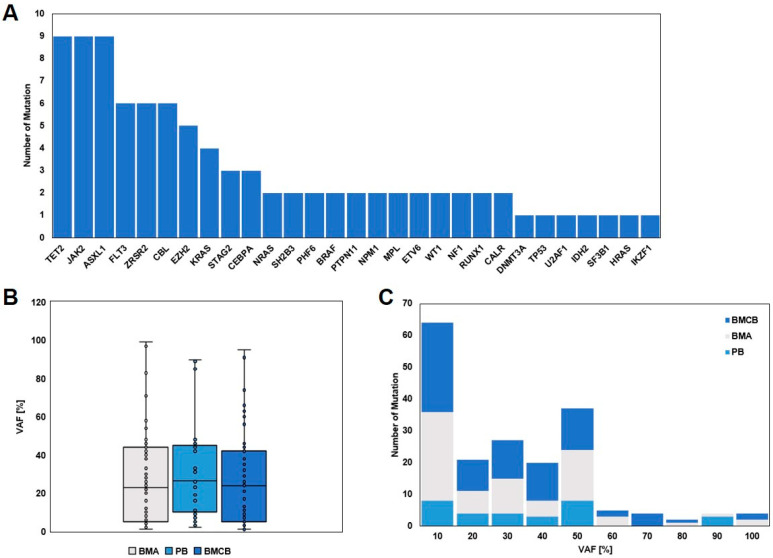
(**A**): Cumulative number of each mutation detected in all cases and in all three specimens (BMA, BMCB, and PB). (**B**): Analysis of VAF of detected mutations depicted by a boxplot showing the distribution and median of VAF of mutations detected in BMA, BMCB, and PB. (**C**): Histogram showing the VAF of mutations and their detection in BMA, BMCB, and PB. Abbreviations: VAF: variant allele frequency, BMA: bone marrow aspiration, BMCB: bone marrow core biopsy, PB: peripheral blood.

Altogether, in all three specimens (BMA, BMCB and PB), in 27 out of 29 cases, 91 mutations could be detected within 29 genes with a median variant allele frequency (VAF) of 24% (range: 1–99%) (Figure 3). The VAF analysis revealed a consistent median VAF across BMA, BMCB, and PB, but with a broad distribution of values. Lower VAF mutations (1–50%) were more frequently detected across all sample types, while higher VAF mutations were predominantly observed in BMA and BMCB only. In five cases, more than one mutation could be found within the same gene (*TET2*, *MPL*, *FLT3*, *WT1*, *ASXL1*, *ZRSR2*, *EZH2*). In two cases in whom BM analysis was performed within follow-up care after successful therapy with MDS and AML (in both, only BMA and BMCB were available), the analysis did not reveal any mutation.

### 3.4. Comparison of the Three Specimens, BMA, BMCB, and PB, and per Case

BMA revealed 74 mutations within 25 genes with a median VAF of 23% (range 1–99%) in 24 cases. The mean number of mutations per case was 2.6 (median 2%, range 0–8%). BMCB showed 85 mutations within 28 genes with a median VAF of 24% (range 1–95%) in 27 cases, which was a mean number of mutations of 2.9 per case (median 2%, range 0–9%). Analysis of the PB of the 12 cases in which this specimen was available found 30 mutations with a median VAF of 26.5% (range 2–90%) in 19 genes and a mean number of mutations per case of 2.5 (median 5%, range 1–8%).

On a case basis, this translated to a gain of information in 11 out of the 29 cases for BMCB, whereas BMCB could only not reveal all mutations detected by BMA in one case. In the remaining 17 cases, there was a complete concordance between both specimens. This difference between BMA and BMCB was statistically significant (*p* = 0.006). Six out of the eleven cases with gain of information for BMCB, the BMA was insufficient (bloody tap), whereas in the seventeen cases with concordance, eleven cases showed a bloody tap.

**Figure 4:**
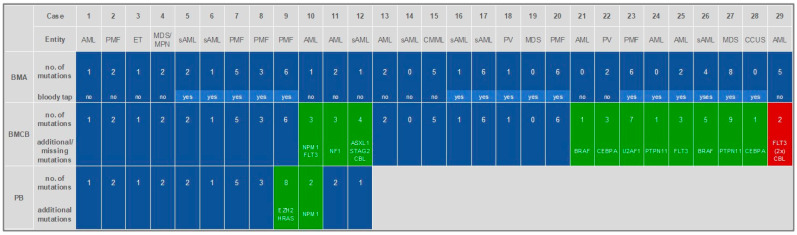
Comparison between BMA, BMCB, and PB on a case basis. 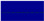 = concordance, 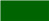 = gain of information, 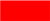 = loss of information. Abbreviations: (s)AML: (secondary) acute myeloid leukemia, PMF: primary myelofibrosis, MDS: myelodysplastic syndrome, PV: polycythemia vera, CCUS: clonal cytopenia of undetermined significance, CMML: chronic myelomonocytic leukemia, ET: essential thrombocytosis, MDS/MPN: myelodysplastic syndrome/myeloproliferative neoplasia—not otherwise specified, BMA: bone marrow aspiration, BMCB: bone marrow core biopsy, PB: peripheral blood, bloody tap: no bone marrow fragments in the aspirate, only blood.

In 12 cases, all three specimens were available for molecular genetic analysis. Full concordance between all three specimens was given in 8 out of these 12 cases, and in 10 out of 12 cases for BMA and PB only. BMCB revealed additional gene mutations compared to BMA in 3 cases. PB could detect altered genes in two more cases, in comparison to BMA. These findings highlight the comprehensive nature of mutation detection when including BMCB, which may capture additional mutations not identified in BMA or PB alone. For detailed information of all specimens and the comparison per case, see Figure 4 and Supplementary Figure S1.

## 4. Discussion

The technical feasibility of molecular genetic analysis of FFPE BMCB has previously been described [9]. However, to the best of our knowledge, this is the first published comparison of BMA, BMCB, and PB in terms of molecular genetic analysis for myeloid neoplasms. Of note, all molecular analyses were performed with the same method, panel, and system. The basis for having available all three specimens (BMA, BMCB, PB) to analyze with the same method in one institution, is that molecular genetic analysis in the diagnosis of hematological diseases is carried out in close cooperation between Hematology and Pathology at the University Hospital Augsburg, as recently published [10].

Our study demonstrated a high level of concordance in mutational analysis across different sample types, with a significant portion of mutations detected in both BMA and BMCB. To ensure the reliability of our sequencing data, we performed comprehensive quality control (QC) analyses for both DNA and RNA sequencing. Specifically, the median values for these parameters indicated robust sequencing performance, with PB and BMCB samples showing less variability and higher consistency compared to BMA samples. The robust QC parameters ensure the accuracy of our findings, and indicate that molecular genetic analysis of FFPE BMCB detects at least the aberrations found in BMA.

The VAF distributions across PB, BMA, and BMCB revealed consistent median values, indicating that the mutations detected in each sample type are present at similar frequencies. However, the broad distribution and variability in VAF values suggest that certain mutations are more heterogeneously distributed within the sampled populations. Notably, lower VAF mutations (1–50%) were more frequently detected across all three sample types, highlighting their pervasive nature. These lower frequency mutations could represent subclonal populations that are important for understanding disease heterogeneity and evolution [11,12]. Higher VAF mutations, although less frequent, were predominantly observed in BMA and BMCB samples. This finding underscores the sensitivity of these sample types in detecting mutations with higher allele frequencies, which are likely to be clonal or near-clonal. The presence of such mutations predominantly in BMA and BMCB suggests that these tissues provide a more representative sampling of the clonal architecture of hematological malignancies. The limited detection of higher VAF mutations in PB may be attributed to the lower cellularity and potential dilution effects in peripheral blood, which can reduce the sensitivity for detecting mutations with higher allele frequencies. Furthermore, the high-quality control (QC) parameters observed for both DNA and RNA sequencing across all sample types reinforce the reliability of our VAF data. This highlights the importance of including BMA and BMCB in diagnostic workflows to ensure comprehensive mutation detection, which is crucial for accurate disease characterization and treatment planning.

Despite the small numbers, our cohort represents the spectrum of the most common myeloid neoplasms with a low accumulation for (s)AML and MPN. The detected mutations reflect the spectrum of the mutation within this entities and is in line with what is known from the literature [9,13,14,15,16]. According to the median VAF of all mutations and specimens with 24% and a range of 1–99%, this is lower than what was published by Palomo et al., with about 30–40% [17]. It should be noted that this cohort was restricted to MDS/MPN entities, which is different from our study, and in our cohort, we had two cases with no mutations at all, which decreased the median VAF. Ultimately, despite the small number of cases, the representativity of our study can be assumed.

In our study, the analysis of the PB showed a concordance in 83% of the cases in comparison to BMA. This high correlation is in line with previously published data for PB and BMA NGS analyses in myeloid neoplasms, underpinning the valid data generated in our analysis [7,8].

Furthermore, BMCB was able to detect additional mutations compared to the BMA in a significant number of cases. The NGS analysis on FFPE BMCB can, therefore, be regarded as technically valid, and it may offer a clinical benefit in addition to BMA and/or PB. The material is usually routinely collected and easily archived as FFPE, and can therefore be analyzed at any time. This can be very useful for clinical routine if the analysis of BMA is or appears inadequate for any reason. In most cases of AML, the analysis of BMA or alternatively PB, is usually sufficiently reliable, as the disease is sufficiently represented in these materials [6,18]. However, this is often not the case with MPNs, and PMF in particular. Bloody tap or dry tap is a common case in these entities [19,20]. A valuable example of this is case 23 with PMF. As expected, the BMA was bloody tap. Nevertheless, mutations were detected in six genes (*JAK2*, *TET2*, *ZRSR2*, *SH2B3*, *EZH2*, *CBL*). A mutation in the *U2AF1* gene was additionally detected in BMCB only. This is relevant because a *U2AF1* mutation is subsumed under the high molecular risk mutations in PMF and included in the corresponding risk scores for PMF [21]. The BMCB may, therefore, enable early and correct risk classification for such a disease.

As described and discussed above, the molecular genetic analysis of BMCB resulted in a significant gain of information compared to BMA, and the question is why this occurred. One reason could be the architecture of the bone marrow with different niches, like the vascular niche, endosteal niche, and reticular niche [22,23]. In these niches, several types of cells are located as hematopoietic stem cells (HSC), multipotent mesenchymal stem cells, nerve fibers, vascular/endothelial cells, and mature blood cells. The interaction of these cells is of great relevance for normal hematopoiesis. In xenograft it was shown, that human HSCs are predominantly located near to trabecular areas in the bone marrow [22]. In case of a malignant disease, the leukemic stem cells are also located in these niches [24,25]. For these reasons, it is quite conceivable that these niches are better represented in the BMCB than in the BMA, which could explain the detection of additional mutations in the BMCB.

There are some limitations in this study. First, the retrospective design and the bias in composition of the cohort. A significant number of cases were caused by the presence of a suboptimal bone marrow aspirate, which prompted the analysis of the peripheral blood as part of the routine, followed by the examination of the bone marrow core biopsy as part of the study. This is why a relatively large number of bloody tap procedures are included in this study. However, particularly in the case of bloody or even dry tap, the analysis of BMCB could be especially important. Another limitation is the small number of cases in this pilot study. But despite this fact, a significant gain of information for BMCB in comparison to BMA could be shown. On the other side, it has to be mentioned that all specimens (BMA, BMCB, and PB) were analyzed on the same platform, so the method of molecular genetic analysis can be ruled out as an explanation for the differences. Another limitation is that for diagnosing myeloid neoplasms, classical cytogenetics is still mandatory. The limitations of RNA sequencing in detecting certain fusion genes, as well as the inability of our targeted amplicon panel to identify classic cytogenetic abnormalities (e.g., monosomy 7, deletions of 5q or 7q). These limitations underscore that BMCB-based NGS complements but cannot replace cytogenetic analysis and RNA-based fusion panels in routine diagnostics.

**Figure 5:**
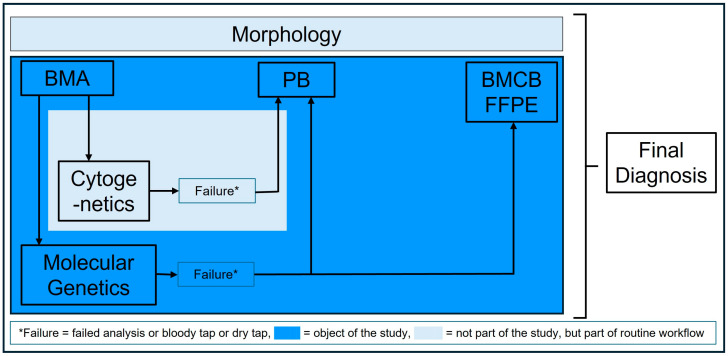
Flowchart of diagnostic procedure including PB and BMCB as an alternative to BMA. Abbreviations: BMA: bone marrow aspiration, BMCB: bone marrow core biopsy, FFPE: formalin-fixed and paraffin-embedded, PB: peripheral blood.

Despite all these limitations, molecular genetic analysis of BMCB could be of additional benefit for diagnosing myeloid neoplasms, as shown in this study. In routine diagnostics, BMA will remain the first choice of specimen, if only because it can be analyzed more quickly than BMCB, which must first be decalcified and embedded in paraffin. However, in the event of an incorrect analysis or bloody tap/dry tap, the data from our study make it possible to fall back not only on PB, as was previously the case, but also on BMCB (see also Figure 5).

## 5. Conclusions

Based on the high level of concordance in the detection of mutations in the different sample types (BMA, BMCB, and PB), our data show that molecular genetic analysis of FFPE BMCB by NGS is technically feasible and a valid tool. At least as many mutations can be detected in BMCB as in BMA, and in some cases, even more information can be expected. In particular, but not limited to, in case of inadequate aspiration or failed analysis of the BMA, BMCB can be used as an optimal supplement, particularly because it can be easily archived as FFPE and is also available retrospectively at any time, not only for routine diagnostics, but also for research.

## Data Availability

All data are provided in the manuscript or the Supplementary Data. Further information can be provided by the corresponding author.

## Supplementary Materials

The following supporting information can be downloaded at: https://www.mdpi.com/article/10.3390/diagnostics15080991/s1, Figure S1: Illustration of all mutated genes in 29 cases detected in BMA, BMCB and PB. Each column represents one case. The number of detected mutations per gene and case is shown accordingly. BMA and BMCB (black, concordant) or in BMCB only (gain = orange). Information loss is highlighted in light red. Abbreviations: *PB* peripheral blood, *BMA* bone marrow aspiration, *BMCB* bone marrow core biopsy; Table S1: List of mutations found.
