# Supplementary figures and images for: Molecular Genetic Analysis of Bone Marrow Core Biopsy as an Alternative or Adjunct to Bone Marrow Aspirate and/or Peripheral Blood in Hematologic Myeloid Neoplasms
Source: Diagnostics (Basel). 2025 Apr 14;15(8):991. doi: 10.3390/diagnostics15080991 (PMC12025941; doi:10.3390/diagnostics15080991)

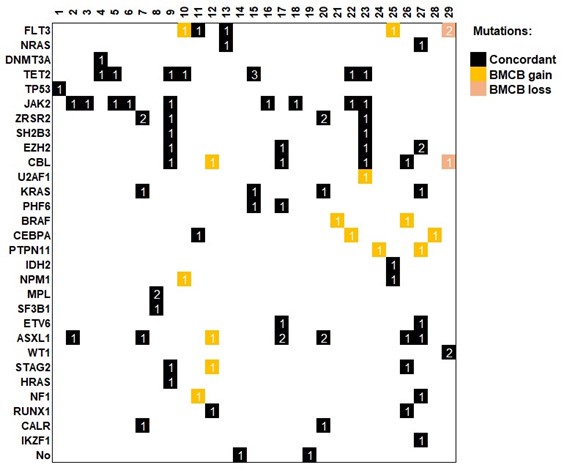

Supplement: Supplementary file 1 [file diagnostics-15-00991-s001.zip › Figure S1.jpg]
